# The Effect of Stochasticity on the *Lac* Operon: An Evolutionary Perspective

**DOI:** 10.1371/journal.pcbi.0030111

**Published:** 2007-06-22

**Authors:** Milan van Hoek, Paulien Hogeweg

**Affiliations:** Theoretical Biology/Bioinformatics Group, Utrecht University, Utrecht, The Netherlands; Washington University, United States of America

## Abstract

The role of stochasticity on gene expression is widely discussed. Both potential advantages and disadvantages have been revealed. In some systems, noise in gene expression has been quantified, in among others the *lac* operon of Escherichia coli. Whether stochastic gene expression in this system is detrimental or beneficial for the cells is, however, still unclear. We are interested in the effects of stochasticity from an evolutionary point of view. We study this question in the *lac* operon, taking a computational approach: using a detailed, quantitative, spatial model, we evolve through a mutation–selection process the shape of the promoter function and therewith the effective amount of stochasticity. We find that noise values for lactose, the natural inducer, are much lower than for artificial, nonmetabolizable inducers, because these artificial inducers experience a stronger positive feedback. In the evolved promoter functions, noise due to stochasticity in gene expression, when induced by lactose, only plays a very minor role in short-term physiological adaptation, because other sources of population heterogeneity dominate. Finally, promoter functions evolved in the stochastic model evolve to higher repressed transcription rates than those evolved in a deterministic version of the model. This causes these promoter functions to experience less stochasticity in gene expression. We show that a high repression rate and hence high stochasticity increases the delay in lactose uptake in a variable environment. We conclude that the *lac* operon evolved such that the impact of stochastic gene expression is minor in its natural environment, but happens to respond with much stronger stochasticity when confronted with artificial inducers. In this particular system, we have shown that stochasticity is detrimental. Moreover, we demonstrate that in silico evolution in a quantitative model, by mutating the parameters of interest, is a promising way to unravel the functional properties of biological systems.

## Author Summary

Gene expression is a process that is inherently stochastic because of the low number of molecules that are involved. In recent years it has become possible to measure the amount of stochasticity in gene expression, which has inspired a debate about the importance of stochasticity in gene expression. Little attention, however, has been paid to stochasticity in gene expression from an evolutionary perspective. We studied the evolutionary consequences of stochastic gene expression in one of the best-known systems of genetic regulation, the *lac* operon of E. coli, which regulates lactose uptake and metabolism. We used a computational approach, in which we let cells evolve their *lac* operon promoter function in a fluctuating, spatially explicit, environment. Cells can in this way adapt to the environment, but also change the amount of stochasticity in gene expression. We find that cells evolve their repressed transcription rates to higher values in a stochastic model than in a deterministic model. Higher repressed transcription rates means less stochasticity, and, hence, these cells appear to avoid stochastic gene expression in this particular system. We find that this can be explained by the fact that stochastic gene expression causes a larger delay in lactose uptake, compared with deterministic gene expression.

## Introduction

Noise in gene expression, i.e., the variation in gene expression in an isogenic population in a homogeneous environment, has drawn much attention in recent years. When two isogenic cells vary in gene expression, this can be due to variation in factors determining gene expression in these cells, such as transcription factors, the concentration of RNA polymerase, the cell cycle, etc., which is called extrinsic noise. When, however, all extrinsic noise is absent, gene expression between these cells would still be different, because gene expression is inherently stochastic, due to the low numbers of molecules involved. The latter is called intrinsic noise.

Indeed, it has been clearly shown that gene expression can be stochastic [1–3]. The implications of stochastic gene expression are, however, much less clear. Stochasticity has been proposed to be deleterious in some systems [4], while being advantageous [5,6] in others. However, there is very little known about the consequences of stochasticity on particular systems.

Maybe the best-known system for genetic regulation is the *lac* operon of E. coli. The *lac* operon codes for three genes, two of which have a function in lactose uptake and metabolism. It codes for a permease protein that transports lactose into the cell and β-galactosidase, which degrades lactose. Gene expression in the *lac* operon has been experimentally shown [2,7] to be stochastic. The *lac* operon is regulated via a positive feedback loop. The operon is induced by allolactose (which is formed by β-galactosidase, from lactose). Induction of the operon again leads to higher permease and β-galactosidase concentrations and hence to higher allolactose concentrations. This positive feedback loop can cause bistability, which means that for certain extracellular inducer concentrations, two stable equilibria exist for the operon, induced and repressed. In a bistable system, stochastic gene expression can cause switching between these equilibria and hence give rise to a bimodal population. Such a bimodal population can be advantageous for the population [5], a phenomenon called bet-hedging.

Bistability for the *lac* operon has been demonstrated using thiomethyl β-D-galactoside (TMG) [8]. Recently, these experiments were repeated and bistability for TMG was confirmed [9]. In this paper, bistability for isopropyl β-D-thiogalactopyranoside (IPTG) and the natural inducer lactose was also tested. Although it is known that IPTG also enters the cell independently from the operon, it still behaved bistably. For lactose, no bistability was found. In [10] we showed that this difference in behavior is because artificial inducers are not degraded by β-galactosidase, while lactose is. Therefore, the positive feedback loop is much stronger for these artificial inducers.

Furthermore, we showed that, using a deterministic evolutionary model of the *lac* operon in a fluctuating environment, cells adapt their promoter function such that the response with respect to lactose becomes continuous instead of bistable [10]. This can be explained by the increase in delay in lactose uptake that bistability unavoidably causes. These in silico evolved promoter functions, however, still behaved bistably with respect to artificial inducers. Indeed, the in silico evolved promoter function resembled the experimentally measured promoter function. Here we study how noise in gene expression influences the adaptation of the *lac* operon promoter function, both on a physiological and an evolutionary time scale.

We use a computational approach to tackle this problem. We modified the previously developed deterministic model [10] for the evolution of the *lac* operon to include stochasticity in gene expression. This model is spatially explicit and consists of cells that grow on glucose and lactose, divide, and die. The intracellular model consists of detailed differential equations describing *lac* operon transcription, translation, and metabolism, with parameters taken from literature. The cells evolve the parameters which determine the *lac* operon promoter function and in this way adapt to the (fluctuating) environment. Importantly, the cells can in this way also adapt to the constraints that are imposed by the fixation of the other parameters. In our view, this is a good way to cope with the inevitable parameter uncertainty. See Materials and Methods for a more detailed description of the model.

We added stochasticity in gene expression on the protein level. We assumed that protein production occurs in bursts, as is experimentally observed [7]. The amount of protein produced per burst (i.e., the burst size) was shown to be geometrically distributed with a mean of five proteins [7]. This observation suffices to make our deterministic model stochastic, as is explained in the Materials and Methods section. Protein degradation is modeled binomially. When a cell divides, the number of proteins is divided between the two cells in a binomial way. In this way we added stochasticity without introducing any unknown parameter.

By comparing the deterministic and stochastic models, we can directly observe the consequences of stochasticity on the *lac* operon, which is experimentally difficult, because the *lac* operon is inherently stochastic. We compared the amount of noise in gene expression in our model with experimentally observed values of noise for the *lac* operon [2] and found that noise in gene expression in our model is comparable to the experimentally observed noise.

These noise values were measured using IPTG. IPTG is not degraded by β-galactosidase and therefore behaves very differently than the natural inducer, lactose. The positive feedback loop is much weaker for lactose than for artificial inducers such as IPTG. Accordingly, we find that noise values for lactose are much lower than for IPTG. Therefore, the effect of stochasticity on evolution of the *lac* operon might be lower than what would be expected from these experiments.

In experiments where stochasticity in gene expression is measured, isogenic populations in well-mixed systems are considered in order to exclude all other sources of population heterogeneity. When we want to investigate the importance of stochasticity in gene expression in natural circumstances, we should, however, also take these other sources of population heterogeneity into account. Therefore, by using a spatially explicit model of cells that evolve their *lac* operon promoter function, spatial and genetic heterogeneity are automatically taken into account. We find that both genetic and spatial heterogeneity contribute more to population heterogeneity than stochasticity in gene expression.

To explore the effect of stochasticity on evolution of the *lac* operon, we compared the results of the evolutionary simulations between the stochastic and the deterministic models [10]. We found that in the stochastic simulations, cells evolve a higher repressed transcription rate than in the deterministic model. Therefore, the promoter functions that evolved in the deterministic model experience more stochasticity when placed in the stochastic model than the promoter functions that evolved in the stochastic model. We show that this causes a reduction of fitness compared with the promoter functions evolved in the stochastic model, due to an increase in delay in lactose uptake. We conclude that in the stochastic model, the promoter functions evolve to minimize stochasticity in gene expression.

Indeed, stochasticity, when growing on lactose, is relatively unimportant for the dynamics of these evolved promoter functions, except sometimes at high glucose, low lactose concentrations. The dynamics when growing on lactose can well be described using a deterministic model. When modeling the dynamics of induction with artificial inducers, stochasticity, however, is much more important, due to the stronger positive feedback, and should be incorporated.

## Results

### Effects of Stochasticity on Noise in Gene Expression

First we studied how stochasticity in gene expression influences noise in gene expression. The promoter function of the cell in large part determines the amount of stochasticity a cell experiences, because stochasticity is higher at low protein levels. Therefore, promoter functions with low repressed transcription rates experience more noise than promoter functions with high repressed transcription rates.

It has been shown that noise in gene expression can be split up into two orthogonal components, intrinsic and extrinsic noise, such that 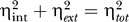 [11]. Here the total noise *η_tot_* is defined as the standard deviation divided by the mean of the population. Extrinsic noise is all noise that would affect two identical, independent copies of one gene in a single cell in exactly the same way, and intrinsic noise is noise that causes differences in expression levels of identical copies in a single cell.

The amount of intrinsic and extrinsic noise of *lac*-repressible promoters in different E. coli strains has been measured [2]. This was done by placing two genes, coding for two fluorescent proteins, which are controlled by identical promoters, in an E. coli strain. The intrinsic and extrinsic noise can now be simultaneously measured, by measuring how the protein levels fluctuate.

**Figure 1:**
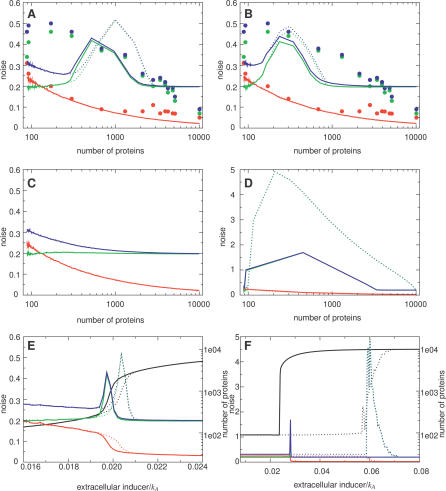
Noise Levels in the Model Compared with the Experimentally Observed Noise (A) Noise as a function of the average number of proteins, for IPTG (k_IPTG_ = 1.35/min). The red lines indicate intrinsic noise, the green lines extrinsic noise, and the blue lines total noise. The filled dots indicate the experimentally observed noise levels, adapted from [2]. Solid lines indicate that cells are initially induced, dotted lines that cells are initially repressed. (B) Noise levels in the model using the higher value for the protein-dependent inducer efflux and k_IPTG_ = 0.10/min. The maximum in the extrinsic noise is shifted to lower protein numbers. (C) Noise levels in the model using lactose as inducer (default model), leading to much lower extrinsic noise. (D) Noise levels in the model using TMG as inducer (k_TMG_ = 0/min), which leads to an increase of the extrinsic noise. (E) Noise as a function of extracellular IPTG concentrations. Inducer concentrations are scaled relative to the binding affinity of the inducer with LacI, the repressor protein of the operon. The black curves indicate the mean protein number. (F) Noise as a function of extracellular TMG concentrations.

We performed similar simulations to validate the stochastic model with these experiments. We initiated a population of 100 induced or repressed cells (solid and dotted lines, respectively, in Figure 1) in a homogeneous environment with a certain extracellular inducer concentration. As in the experiments, spatial and genetic heterogeneity are absent. We waited for 41 hours, more than enough time for cells to go to equilibrium in the deterministic model. Then we calculated the noise in the protein number in the population. Noise levels were obtained in the same way as in the experiments. We kept track of the activity of two independent, identical *lac*-repressible genes that do not have a function in lactose uptake.

Because we did not introduce any free parameter in the model when introducing stochasticity, noise levels only depend on the promoter function used. We used a promoter function that has the same repression rate as the one used in the experiments (≈110, see Figure 1A). As a comparison, for the wild-type *lac* promoter function, the repression rate has been reported to be 170 [9]. Furthermore, we used a Hill-coefficient of 4.0 [12]. For the growth rate, we took 0.01/min in these simulations. The induced transcription rate of the operon in these simulations is equal to the maximal transcription rate we imposed during the evolutionary experiments.

The experiments were done using IPTG, an artificial inducer of the *lac* operon. In contrast to lactose, IPTG is not degraded by β-galactosidase. This changes the dynamics, hence the noise, of the system considerably. Our model describes operon dynamics with lactose as inducer. Using parameters for IPTG, as used in [13], and not allowing for degradation of IPTG by β-galactosidase, our model can also describe *lac* operon dynamics when induced by IPTG. It is known that IPTG also enters the cell in the absence of permease. This is not taken into account in our model describing lactose dynamics and hence we add a permease-independent influx term for IPTG (see Materials and Methods). The amount of permease independent influx we chose is such that the maximum in the total noise is similar to that in the experiments.

In Figure 1A the results for IPTG are shown, using a permease-independent influx, k_IPTG_, of 1.35/min. The intrinsic noise found in our simulations is almost identical to the experimentally observed intrinsic noise. Intrinsic noise levels in the model only depend on the number of proteins. We found that the data can almost perfectly be fitted by the function *η*_int_ = C*P^a^, with *a* = −0.505, and *C* = 2.35 with an *R* ^2^-value of 0.99996. At steady state, the theoretical prediction [14] is *a* = −0.5 and 
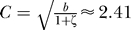
 , where *b* equals the average burst size and *ζ* is the ratio of mRNA to protein lifetimes. This figure also confirms that the induced transcription rate we used has the right order of magnitude. It has been reported that the maximal in vitro transcription rate is approximately 0.18/min, but that the maximal in vivo transcription rate is approximately 1–10/min [15]. In contrast to [16], who used the maximal in vitro transcription rate, we used a maximal transcription rate of 11/min.

Qualitatively, the extrinsic noise corresponds with the experimentally observed noise. The maximum in the extrinsic noise is caused by the positive feedback loop in the *lac* operon. Fluctuations due to the intrinsic noise are amplified by the positive feedback, but only at intermediate inducer (hence protein) concentrations, where the promoter function is steepest. Note that the cell cycle and the intracellular inducer concentration are the only extrinsic noise sources we included in the model.

The maximum in the extrinsic noise is located at lower protein concentrations in the data than in our model. This is because the extrinsic noise is high if the positive feedback is strong. Sufficient positive feedback can only be accomplished if the protein-dependent and protein-independent inducer influx are of the same order of magnitude. Therefore, when the protein-independent inducer influx is high, the maximum in the extrinsic noise will be located at high protein concentrations. We can reproduce the experimental data better if we assume a lower growth rate or a higher protein-dependent inducer efflux. Both these changes diminish the positive feedback and therefore we need a smaller protein-independent influx to fit the maximum amount of extrinsic noise. Therefore, the maximum in extrinsic noise will also be shifted to lower protein concentrations. When we use, for example, a protein-dependent inducer efflux of 300 mM/(mM permease min) instead of 49.35, the value reported by [13], we indeed find that the maximum in the extrinsic noise shifts to lower protein numbers (Figure 1B.) We obtained these curves for a protein-independent inducer influx (k_IPTG_) of 0.1/min.

For lactose as inducer, the picture is very different (Figure 1C). The intrinsic noise remained unchanged, but extrinsic noise changed considerably. There is still a maximum in extrinsic noise, but this is barely visible. The reason is that the positive feedback loop is much weaker for lactose than for IPTG, due to the degradation of lactose by β-galactosidase. Indeed, the operon used is monostable for lactose. For lactose, we found that extrinsic noise is almost completely determined by the cell cycle for all protein numbers, instead of only for high protein numbers as we found for IPTG. We conclude that the results of the experiments can only be understood when we realize that IPTG was used as an inducer. When lactose as inducer is used, extrinsic noise levels as high as in the experiments can in our model only be observed when the repressed transcription rate is considerably lower.

Finally, we tried to simulate noise in gene expression for TMG, another artificial inducer. As IPTG, TMG is not degraded by β-galactosidase, but in contrast to IPTG, there is no permease-independent influx. Therefore, the positive feedback loop is stronger for TMG. We simulate TMG by using the same parameters as for IPTG, except the permease influx rate is changed from 1.35/min to 0/min. We observe that the extrinsic noise increases drastically (Figure 1D), while the intrinsic noise again remains unchanged. Indeed, the stronger positive feedback loop increases extrinsic noise.

Experimentally, it has been observed that the wild-type *lac* operon behaves bistably for both IPTG and TMG [9]. For IPTG, however, bistability is expected to be much less severe [9]. We find that the experimentally observed noise can best be described by a promoter function that is just bistable for IPTG. This can be seen when we compare the amount of noise for initially induced and repressed cells. Although all individual cells are in equilibrium, the results are different when we start with an initially induced population or an initially repressed population. For TMG, we also find that the promoter function is much more bistable.

To observe the effect of bistability better, we also show how noise levels depend on the extracellular inducer concentration, for both IPTG and TMG (Figure 1E and 1F). The hysteresis-loop, indicating that for certain extracellular inducer concentrations the amount of proteins is dependent on the history of the cells, is clearly visible. Indeed, for TMG this loop is much larger than for IPTG. Furthermore, we see that when inducer concentrations are low, transitions from the induced to the repressed equilibrium are more likely, and vice versa.

### Effects of Stochasticity on Population Heterogeneity

In the previous section, we showed that stochasticity in gene expression can cause significant amounts of noise when the operon is repressed. The amount of noise at intermediate inducer concentrations was, however, much larger for IPTG and TMG than for lactose.

These experiments and simulations were done using cells with identical promoters in a well-mixed environment. In natural and evolving populations, different cells will have slightly different promoter functions, due to genetic variation, and the environment will not be well-mixed [17,18]. Both these factors can cause population heterogeneity, but are neglected in almost all experiments. This makes sense if we want to study whether stochasticity in gene expression occurs at all. Stochasticity in gene expression can only be proven when gene expression is different in two identical cells in an identical environment. For the importance of stochasticity in natural circumstances, however, other factors causing population heterogeneity need to be considered.

We embedded the intracellular stochastic model of *lac* operon dynamics in an evolutionary, spatial context, as described in the Materials and Methods section and in more detail in [10]. We used this stochastic model and the deterministic model [10] to unravel the contributions of the various factors on population heterogeneity.

**Figure 2:**
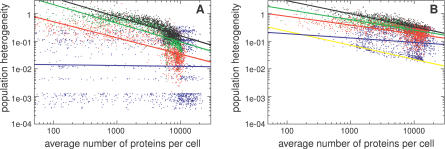
Population Heterogeneity As a Function of Protein Number, Both for the Deterministic and Stochastic Simulations (A) Population heterogeneity in the deterministic simulations, expressed as standard deviation/mean of the number of β-galactosidase proteins per cell. Different colors represent different simulations. All dots indicate the population heterogeneity at certain equally spaced timepoints. Solid lines give the result of a power-law regression between these dots. Black: an evolutionary simulation in a spatial environment, both genetic and spatial heterogeneity, evolutionary timescale. Red: an evolutionary simulation in a well-mixed environment, genetic heterogeneity, but no spatial heterogeneity, evolutionary timescale. Green: a simulation with one clone (the last common ancestor of the “black” simulation), spatial heterogeneity, but no genetic heterogeneity, physiological timescale. Blue: a simulation with one clone (the last common ancestor of the “red” simulation), no spatial heterogeneity, no genetic heterogeneity, physiological timescale. (B) Population heterogeneity in the stochastic simulations. All colors are as in Figure 2A, except yellow: regression curve of intrinsic noise versus average protein number (from Figure 1).

To this end, we performed evolutionary simulations in both a spatial and a well-mixed environment, for both the stochastic model and the deterministic model. In these simulations, cells evolve their promoter function in an environment in which the glucose and lactose concentrations fluctuate. To understand the effect of genetic diversity, we also performed simulations using a genetically identical population. We used the last common ancestor (the last individual cell that had the whole population at the end of a simulation as offspring) of each evolutionary simulation for this. In this way, we can compare the effects when genetic variation, spatial variation, both, or none are incorporated, in both the deterministic model (Figure 2A) and the stochastic model (Figure 2B), yielding eight different simulations.

Of the four sources of population heterogeneity in our model, spatial, genetic, stochastic, and cell cycle related, we can exclude all, except for the cell cycle. When all these three noise sources were excluded, we still observed some population heterogeneity, but it was completely independent of protein number (Figure 2A, blue dots). The population heterogeneity varied wildly over time, due to the partial synchronization of the cells. Sometimes all cells have just divided, and population heterogeneity is very low. When only half of the population has recently divided, population heterogeneity is maximal. This explains the extremely broad distribution of blue dots in Figure 2A.

When genetic or spatial heterogeneity was present, very low values of population heterogeneity did not occur anymore (Figure 2A, red, green, and black dots). When these sources of population heterogeneity were present, population heterogeneity became inversely correlated with protein number. It is clear that in our model, space has a larger effect on population heterogeneity than genetic variation (see Figure 2A, red and green dots).

When we compare population heterogeneity in the deterministic model (Figure 2A) with the population heterogeneity in the stochastic model (Figure 2B), we find, as expected, the largest difference when spatial and genetic heterogeneity are both absent. Intrinsic noise (Figure 2B, yellow line) gives a lower boundary to the population heterogeneity in the stochastic model. The mean population heterogeneity is increased considerably by stochastic gene expression. When genetic or spatial heterogeneity was present in the stochastic model (Figure 2B, red and green), we observed, however, that much of the difference in population heterogeneity between the deterministic model and the stochastic model disappeared. Population heterogeneity due to stochastic gene expression, therefore, apparently is small compared with population heterogeneity due to spatial and genetic variation.

Intrinsic noise, as shown in the previous section, follows power law behavior with respect to protein number, with a coefficient of 0.5, just like Poisson noise. Surprisingly, both in the deterministic model (Figure 2A) and the stochastic model (Figure 2B) we see that if spatial or genetic variation is present, the data can still reasonably be described using a power law. In Table 1, we give the regression and correlation coefficient of a power law regression of the data.

**Table 1:**
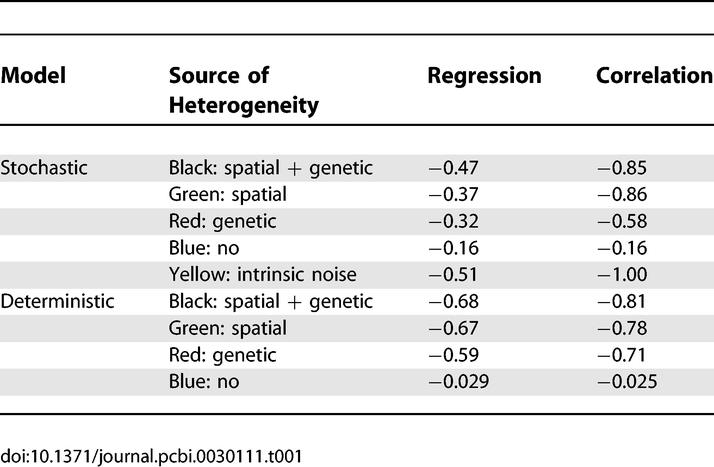
Regression and Correlation Coefficients

The regression coefficient indicates how strong population heterogeneity and protein number are correlated. The more sources causing population heterogeneity, the higher the regression coefficient is. Genetic heterogeneity correlates with protein number because the selection pressure on the induced operon is higher than on the repressed operon. The reason for this is that the cost for promoter activity is much more important for the induced operon than for the repressed operon. This appears to be a reasonable assumption that is likely to hold in the natural environment of E. coli.

Population heterogeneity due to space is largest at intermediate extracellular inducer concentrations. At very high inducer concentrations, noise in the inducer concentrations does not cause noise in gene expression, because of the sigmoid shape of the promoter function. This also holds for very low extracellular inducer concentrations. For intermediate inducer concentration, gene expression is very much influenced by spatial heterogeneity. We observed, however, a monotonic relationship between protein number and noise due to spatial heterogeneity. This is because when the promoter activity of cells becomes low, cells stop lactose consumption, and therefore cells never experience very low lactose concentrations. This effect is likely to play a role in the natural environment, but in the natural environment, lactose degradation is probably not only due to *E. coli,* and we might expect noise due to spatial heterogeneity not to decrease monotonically with protein number.

From all this we expect that only in a monomorphic population, living in a well-mixed environment, does stochasticity in gene expression play an important role. Whether stochasticity influences evolution, however, is therefore doubtful. To study whether stochasticity does or does not play an important role in evolution, in the next section we compare evolution of the *lac* operon in the stochastic and deterministic models. We do this in a well-mixed environment, because there stochasticity is expected to have the largest influence.

### Effects of Stochasticity on Evolution in a Well-Mixed Environment

**Figure 3:**
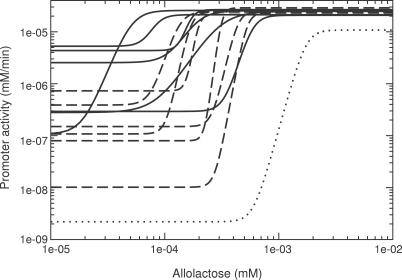
Promoter Functions at Zero Glucose, Evolution in a Well-Mixed Environment Dotted line, initial promoter function. Solid lines, promoter functions of the six last common ancestors of the stochastic evolutionary simulations. Dashed lines, promoter functions of the six last common ancestors of the deterministic evolutionary simulations.

We performed six independent evolutionary simulations in well-mixed environments, in both the deterministic model and the stochastic model. For all 12 last common ancestors, we plotted the promoter activity if no glucose is present (Figure 3). Most of the changes between the initial promoter function (dotted line) and the evolved promoter functions are consistent between all simulations. The induced transcription rate is increased, to approximately the same value in all 12 simulations. The steep part of the promoter function evolved to approximately 10-fold lower allolactose concentrations. Finally, the repressed transcription rate increased very significantly. As proven in [10], the repressed transcription rate in large part determines whether a promoter function is bistable. The initial promoter function was chosen to be bistable. Of the 12 last common ancestors, only one was bistable. This is indeed the promoter function with the lowest repressed transcription rate. There appears to be a trend that promoter functions, which evolved in the stochastic simulations, have higher repressed transcription rates than those evolved in the deterministic simulations.

To check this more precisely, we calculated the time average of the repressed transcription rate at zero glucose for all 12 simulations. There is considerable variation in this quantity over time during the whole evolutionary simulation.

The first quarter of the evolutionary simulation was not taken into account, to give the cells time to adapt somewhat to the environment. We found that on average in the stochastic simulations the repressed transcription rate was 5.5-fold higher than in the deterministic simulations. In the stochastic simulations, we found an average repression rate of approximately 45, while for the deterministic model this was approximately 250. Previously, a repression rate of approximately 170 was experimentally found [9].

Whether this difference is significant, we checked by permuting the average repressed transcription rates over the different simulations and calculating the difference between the stochastic and deterministic simulations for all possible permutations. In less than 3% of the permutations, we found a larger average difference than observed, which gives a measure of the significance of this result.

Next, we performed competition experiments between two promoter functions, one evolved in the stochastic model, the other in the deterministic model. We used the same environment as was used for the evolutionary simulations. For clarity we chose the promoter function with the highest repressed transcription rate and the promoter function with the lowest repressed transcription rate in Figure 3. Both these promoter functions perform well in comparison with the five other evolved promoter functions.

We placed these two promoter functions in the stochastic and deterministic model. When one population died out, we stopped the simulation and scored which promoter function had died out. No mutation was allowed during these competition experiments. Both for the stochastic model and for the deterministic model, we performed 100 of these competition experiments. In the deterministic model, we found that in 61 cases the promoter function with the low repressed transcription rate won, while in the stochastic model in 60 cases the promoter function with the high repressed transcription rate won. Using a two-tailed binomial test, this corresponds to a *p*-value of 0.057 and 0.035, respectively. Combined, this gives a *p*-value of 0.0020. This confirms that the promoter with the low repressed transcription rate performs better in the deterministic model, while the promoter with the high repressed transcription rate performs better in the stochastic model. Due to the small population sizes during the simulations (on average approximately 200), there is a lot of drift. This causes the most competitive promoter function to not always win the competition experiment.

**Figure 4:**
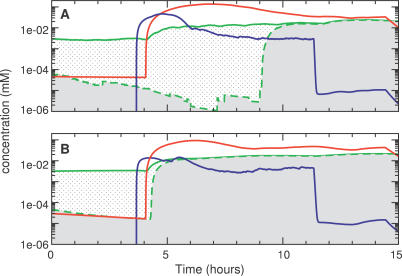
Dynamics of Two Promoter Functions (A) The stochastic model: red indicates external lactose concentration; blue, external glucose concentration; and green, the β-galactosidase concentration (which is converted to mM to compare it with the deterministic model, a concentration of 2e-06 mM corresponds with 1 molecule per cell, just after cell division). The solid line indicates the β-galactosidase concentration for the promoter function with the high repressed transcription rate, while the dashed line corresponds to the promoter function with the low repressed transcription rate. (B) Same as (A), for the deterministic model. Note that, because cells behave differently in each model, the extracellular glucose and lactose concentrations are not identical (but very similar) in both (A) and (B).

To understand this result, we studied the dynamics of these two promoter functions in both the deterministic model and the stochastic model. For both models, we again initiated a heterogeneous population and followed the dynamics of two individual cells, one with a high and one with a low repression rate. In both models, we used an identical environment, such that we can compare the dynamics. An example of the dynamics during a period of lactose influx is shown in Figure 4. Figure 4A shows the dynamics of both promoter functions in the stochastic model, while Figure 4B shows the dynamics in the deterministic model, in the same environment.

The promoter function with the high repressed transcription rate behaves almost identically, whether placed in the stochastic or the deterministic model. The only observable difference is caused by the cell cycle. For the promoter function with the low repressed transcription rate, the picture is very different. This promoter function is slightly bistable. Only when the external lactose concentration exceeds approximately 0.02 mM, does the operon switch on. This causes a slight delay in protein production, as can be seen in Figure 4B. In the stochastic model, the delay is, however, much larger. Only after approximately five hours, does the operon switch on. Because protein production occurs in bursts, and not in a gradual way, the cell has to wait for a sufficiently large burst to become induced. It is important to note that for the lactose concentrations the cells experience after approximately 4.5 hours, the operon is not bistable in the deterministic model, but, nevertheless, in the stochastic model the operon is not able to switch to the induced state.

Figure 4 only shows the dynamics during one lactose pulse. In the evolutionary and competition experiments, many different pulses, of different height and length, are experienced. Also, the periods without influx have different lengths. Therefore, for every pulse the situation is somewhat different. During the periods without lactose influx, the promoter with the low repressed transcription rate has a higher growth rate than the promoter with the high repressed transcription rate, because its cost for operon activity is lower. When lactose influx starts, the operon with the high repressed transcription rate has a higher growth rate, because it can start lactose uptake earlier.

**Figure 5:**
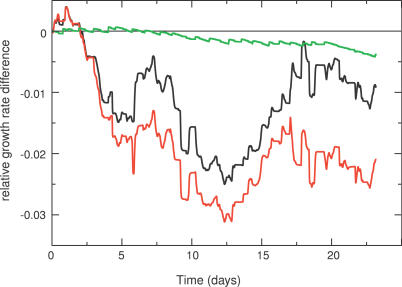
Growth Rate of the Two Promoter Functions in Both Models Here we report the growth rate difference compared with the promoter function with the low repressed transcription rate in the deterministic model. Black, promoter function with the high repressed transcription rate in the stochastic model. Red, promoter function with the low repressed transcription rate in the stochastic model. Green, promoter function with the high repressed transcription rate in the deterministic model.

Therefore, depending on the state of the environment (whether there is lactose influx or not), the growth rate difference between the two promoters will be positive or negative. In an environment with the same parameters as the environment used for the evolutionary and competition experiments, we kept track of the growth rates during a period of time approximately equal to the time that is needed for one competition experiment. The result is shown in Figure 5. The green line indicates that the promoter function with the high repressed transcription rate on average has a lower growth rate in the deterministic model than the promoter function with the low repressed transcription rate, although the growth rate difference indeed fluctuates, according to whether lactose is present in the environment or not. Again this proves that the promoter function with the low repressed transcription rate performs better in the deterministic model. When we compare both promoter functions in the stochastic model (the black and the red line in Figure 5), we see, however, that the average growth rate of the promoter function with the high repressed transcription rate is highest.

This all shows that promoter functions evolved in the stochastic simulations evolve to a higher repressed transcription rate, which make them outcompete promoter functions with very low repressed transcription rates. In the deterministic simulations, the situation is the opposite, and these high repressed transcription rates are not optimal.

### The Importance of Nonequilibrium Conditions

Whether the cell is in equilibrium has considerable effect on the noise levels. In this section, we discuss two examples in which nonequilibrium dynamics is crucial for the amount of noise.

Bistability in a deterministic system causes hysteresis. Depending on the history of the system, the system will be in one of the two equilibria. This hysteretic behavior disappears when stochasticity is added. In relatively short time scales, the system remains hysteretic, i.e., when cells were induced, they remained induced, while when cells were repressed, they remained repressed. In longer time scales, transitions between the two equilibria are possible. Therefore, the probability distribution of the state of cells goes to a stable equilibrium, with some cells in the repressed and others in the induced state, depending not on the history but on the transition probability between the two equilibria. Therefore, if we would have waited long enough, the difference in noise levels between initially repressed and induced cells as shown in Figure 1 would have disappeared. Most of the cells would have gone to the induced state, because the transition probability to go from the induced to the repressed state is lower than vice versa, due to the lower noise for the induced operon.

**Figure 6:**
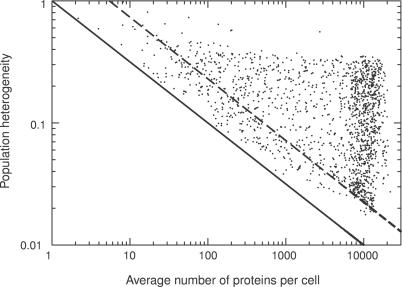
Population Heterogeneity Can Be Lower Than the Intrinsic Noise One promoter function is placed in a well-mixed environment; therefore, both genetic and spatial heterogeneity are absent. Dashed line, intrinsic noise (from Figure 1): 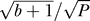 , with *P* the number of proteins in the cell. Solid line, Poisson noise: 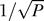 .

A second example is shown in Figure 6. Here we show the population heterogeneity of a promoter function belonging to a last common ancestor of an evolutionary simulation. Both genetic and spatial heterogeneity are absent. We observe that the population heterogeneity is frequently lower than the intrinsic noise. Intrinsic noise is in general seen as inherent and therefore unavoidable for a cell. We would expect then that noise cannot be lower than the intrinsic noise. Here we see, however, that this is not necessarily so.

This striking observation can be understood when we realize that the intrinsic noise was measured in cells that were in equilibrium. During our simulations, cells were very often not in equilibrium. Intuitively, we might expect that cells that are not in equilibrium have even higher population heterogeneity, but this is apparently not the case. If cells have a much higher protein concentration than the equilibrium value (if, for example, external lactose has just been depleted), the protein concentration decreases. In such a situation, transcription can be neglected and the dynamics are purely determined by protein degradation and dilution. Noise caused by protein degradation and dilution is, however, much lower than transcriptional noise, because degradation does not occur in bursts. Noise due to degradation and dilution can be described as 
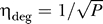
 , Poisson noise (solid line in Figure 6). Translational noise is, however, much broader than Poisson: 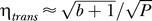 (dashed line in Figure 6), where *b* is the burst size. Indeed, we observe that Poisson noise does give a lower boundary to the population heterogeneity, whereas the intrinsic noise does not.

## Discussion

We study the influence of stochasticity in gene expression on evolutionary adaptation of the *lac* operon of E. coli. To this end, we used a detailed quantitative model of the *lac* operon in which stochasticity is incorporated on the protein level. This approach has the advantage that only one (experimentally known) parameter needs to be added to the model to make it stochastic, namely the average burst size of protein translation. We find good agreement between noise levels in our model and experimental noise measurements [2].

The experimentally observed noise levels, however, can only be explained when we realize that IPTG, which is not degraded by β-galactosidase, is used as inducer. We find that induction by IPTG leads to very different dynamics than induction by the natural inducer lactose (see also [10]). When the operon is induced by lactose, stochasticity in gene expression is strongly reduced, and the total noise is mostly determined by the cell cycle. This is due to the fact that degradation of lactose by β-galactosidase reduces the strength of the positive feedback loop. Induction by TMG, however, leads to even higher noise values, because in contrast to IPTG, there is no protein-independent TMG influx. It would be very interesting to measure noise in gene expression of the *lac* operon, for TMG, IPTG, and lactose, to validate these results.

In literature, different values for the Hill-coefficient are reported. For example, in [9] a Hill-coefficient of 2 is used, while in [12] a value of 4.0 was measured. When the Hill-coefficient is high, the positive feedback is strong and noise values are high. For IPTG, however, the noise curves would be very similar, because we chose the protein-independent inducer influx such that the maximal amount of noise corresponded to the experimentally measured noise values. For lactose we found that, all other parameters being equal, the promoter function only becomes bistable when the Hill-coefficient is larger than 52, which is clearly unrealistically high. During the evolutionary experiments, the Hill-coefficient can be mutated and it mostly varies between 2 and 10.

To investigate the effect of different noise sources on population heterogeneity, we compared the amount of population heterogeneity in simulations with and without space, mutations, and stochasticity in gene expression. In these simulations the operons were induced by lactose, instead of by artificial inducers. We observed that only in the well-mixed simulations, without mutation, was the amount of population heterogeneity much larger in the stochastic than in the deterministic simulation. If spatial or genetic heterogeneity was added, stochasticity hardly influenced population heterogeneity. Surprisingly, we found that both genetic and spatial heterogeneity decrease with protein number, more or less in the same way as population heterogeneity by stochastic gene expression. Especially for genetic heterogeneity, we expect this also to be the case in nature.

In nature, it seems likely that the spatial heterogeneity is very large. The gut is a highly diverse ecosystem [18], and not at all well-mixed. It has been shown that E. coli is able to entirely change its *lac* operon promoter function in a few hundred generations [17]. This suggests that in nature the genetic diversity is also high. Therefore, we believe that the large genetic and spatial differences in our model are biologically realistic. However, we did check our results for a ten times lower mutation rate and ten times higher diffusion constant, which determine genetic and spatial heterogeneity, respectively. Even when both the mutation rate and the diffusion constant are modified, we observe that noise due to stochastic gene expression is only comparable to spatial and genetic heterogeneity at very low protein numbers (unpublished data).

Finally, we directly compared the promoter functions evolved in the deterministic and the stochastic evolutionary simulations. We observed that in the stochastic simulations cells evolve to higher repressed transcription rates and thus prevent stochasticity in gene expression. We show that this is because in the stochastic model low transcription rates cause longer delays than in the deterministic model.

This is in striking contrast with the result found in [6]. There it was found that in *Saccharomyces cerevisiae,* bursts in gene expression enable a more rapid cell response. When we initialize cells in an environment with a fixed inducer concentration, for which the operon is bistable, we also see that larger bursts enable a more rapid response to this inducer concentration than smaller bursts. Indeed, in the deterministic model, cells would stay indefinitely in the “wrong” equilibrium. Because in our model, however, the inducer concentration varies over time, the inducer concentration quickly increases over the point at which the operon is still bistable, and in the deterministic model the cells then respond very fast. In the stochastic model, cells still need to wait for a sufficiently large burst to occur even when the inducer concentration has increased over the concentrations for which the cells are bistable. Even when the average burst size is large, this takes a long time, because then the frequency of the bursts is lower. This explains why the net effect of stochasticity in our model is negative for such promoters (compare the growth rate of the promoter function with the low repressed transcription rate in the deterministic and the stochastic models; Figure 5, red line). Furthermore, having a somewhat higher repressed transcription rate ensures that *all* cells have a rapid response.

Both in the deterministic and the stochastic simulations, bistability with respect to lactose is most often avoided (except at high glucose concentrations, which are very rare). Although the promoter function evolved in the deterministic model, which we used in Figure 4, is slightly bistable, this has little influence on the behavior of this promoter function in the deterministic model, while the behavior in the stochastic model is changed drastically by the bistability.

In [10] we already showed that when using a 10 times higher cost for *lac* operon activity, or a different environment (with longer or shorter periods with and without lactose), bistability was also avoided. In the stochastic model, we also performed simulations in different environments, but again the results did not change essentially.

We conclude that stochasticity cannot avoid the inherent disadvantages of bistability (namely longer delays in protein dynamics [10]). Even more, we showed that bistability is even more deleterious in the presence of stochastic gene expression than in a deterministic system. These conclusions are in line with [4], whose authors have shown that essential genes in S. cerevisiae have evolved to lower noise values than nonessential genes and thus that stochasticity for many genes appears to be detrimental during evolution.

## Materials and Methods

In this section we discuss the computational model that is used in this paper. We used both a deterministic and a stochastic version of the model. The deterministic version is explained in detail in [10], but here we describe the major points of the deterministic model. For the stochastic version of the model, we modified this deterministic model in a few ways. These modifications we describe in detail.

### Model for simulating *lac* operon evolution.

The deterministic model is a spatially explicit, computational model of E. coli cells, growing on glucose and lactose while evolving their *lac* operon promoter function. It consists of an intracellular and an extracellular part.

Intracellular dynamics. The intracellular dynamics is modeled using ten differential equations, following [19]. The following intracellular variables are incorporated: mRNA (M) β-galactosidase (B), permease (P), lactose (L), allolactose (A), glucose (G), glucose-6-phosphate (G6P), cAMP (C), ATP (ATP), and cell size (X). Transcription of the *lac* operon, translation, lactose and glucose uptake and metabolism, cAMP and energy production, and cell growth are all modeled in detail. When possible, parameter values are taken from literature. All ten differential equations are integrated using a timestep of 0.2 seconds. Here we shortly discuss all differential equations. A list of all parameters is given in Table S1.

Transcription is modeled as a two-dimensional Hill-function, dependent on the cAMP and allolactose concentration (see [12]). In this way, glucose, via cAMP, represses the operon, while lactose, via allolactose, induces the operon. This two-dimensional Hill-function depends on 11 biochemical parameters, such as the k-value and Hill-coefficient of allolactose binding to the repressor. In the evolutionary simulations, these are the only parameters that can mutate, all other parameters are fixed, because we are interested in the evolution of the promoter function, given realistic boundary conditions (which are the other parameters in the intracellular model).

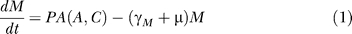


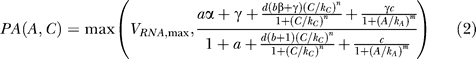



*a* = *RNAP/k_RNAP_*, RNA-polymerase in units of its dissociation constant for binding to a free site.

*b = RNAP/k_RNACP_*, RNA-polymerase in units of its dissociation constant for binding to a site with bound CRP (cAMP receptor protein).

*c = LACI^T^/k_LACI_*, the total LacI concentration in units of its dissociation constant for binding to its site.

*d = CRP_T_/k_CRP_*, the total CRP concentration in units of its dissociation constant for binding to its site.

α, the transcription rate when RNA Polymerase is bound to the DNA, but CRP and LacI are not.

β, the transcription rate when both RNA Polymerase and CRP are bound, but LacI is not bound to the DNA.

γ, “leakiness,” the transcription rate when RNA Polymerase is not bound to the DNA.

*k_A_*, k-value for allolactose binding to LacI.

*m*, Hill-coefficient describing cooperativity in binding of allolactose to LacI.

*k_C_*, k-value for cAMP binding to CRP.

*n*, Hill-coefficient describing cooperativity in binding of cAMP to CRP.

Protein production (β-galactosidase and permease) depends on the mRNA concentration, and proteins are slowly degraded.

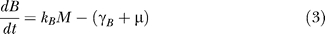


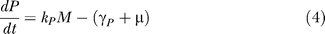



Lactose influx is permease-dependent, while lactose degradation and conversion to allolactose is dependent on β-galactosidase. Small protein-independent lactose and allolactose degradation terms also are added.

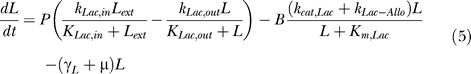


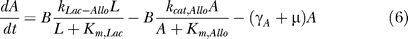



Lactose is converted to glucose by β-galactosidase. Glucose uptake from the medium also is incorporated. Glucose metabolism (via glycolysis and TCA-cycle) produces ATP on which the cells grow. ATP production depends on the glycolytic fluxes and the fluxes through the TCA-cycle. ATP is consumed by basal metabolism, cell growth, and *lac* operon activity. The cAMP concentration is assumed to be dependent on the glucose influx rate (see [19]).

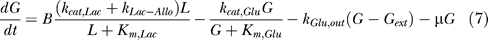


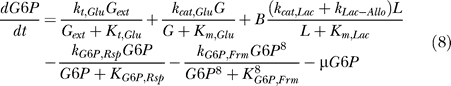


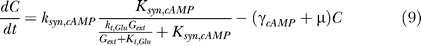


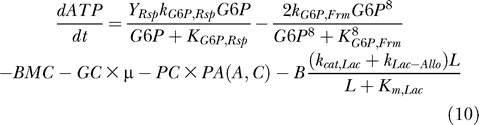


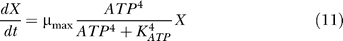



*The spatial model*. Cells, of which the dynamics are determined by the above-described intracellular model, are placed on a square grid of 25 × 25 grid points. These cells grow on extracellular glucose and lactose. The extracellular glucose and lactose concentrations are determined by a fluctuating influx of glucose and lactose into the grid, consumption of glucose and lactose by the cells, and diffusion over the grid. In the well-mixed simulations, we assume infinite diffusion, such that the glucose and lactose concentrations are equal over the whole grid. Furthermore, the cells are shuffled randomly over the grid.

Glucose and lactose influx into the grid is modeled in pulses, independently of each other. Pulses of glucose and lactose influx both have an average duration of 11 hours. The total amount of carbon influx is on average equal for each pulse, such that short pulses have high influx rates, and vice versa. Every pulse has a probability of 10% of being instantaneous, such that very high glucose or lactose concentrations also sometimes occur. In 25% of these cases, simultaneous glucose AND lactose influx occurs, in order to enforce simultaneous high glucose AND lactose concentrations. These pulses are followed by a period without glucose or lactose influx, also on average 11 hours.

This environment is chosen for two reasons. First, all combinations of glucose, lactose, glucose AND lactose, and neither one occur repeatedly. Second, the average length of the periods is chosen such that cells can just adapt their protein concentrations to the environment.

The cells consume glucose and lactose, grow (as the growth rate is given by the intracellular model), divide (if their size has doubled and there is space to reproduce), and move randomly over the grid. The cells are diluted in a density-dependent way, and cells “die” if they have zero energy (although this happens very rarely). Therefore, fitness differences are mainly caused by differences in growth rates between cells (which are again dependent on glucose and lactose uptake) instead of by differences in death rates.

In the evolutionary experiments, we always start with a monomorphic population of approximately 60 cells that are bistable with respect to lactose and have a diauxic shift (such that glucose and lactose are consumed sequentially). After each cell division, the daughter cell has a probability of 0.01 to mutate one of the 11 biochemical parameters that determine the shape of the *lac* operon promoter function. All other parameters are kept constant. This means that cells can adapt the precise shape of the promoter function to the (fluctuating) environment. We let the cells evolve for approximately 2,000 days, which is sufficient to adapt to the environment.

#### Modification of the model to incorporate stochasticity.

It has been predicted [20] and recently experimentally verified [7] that protein production occurs in bursts and that the size of these bursts, i.e., the number of proteins that are produced per burst, is geometrically distributed. The reason for this is that mRNA molecules are translated several times, before they are degraded. This leads to the rapid production of several proteins once an mRNA molecule is produced, and protein production ends when the mRNA molecule is degraded. When an mRNA molecule is translated, it cannot be degraded. Therefore, after each translation, the mRNA molecule has a probability p to be translated again and a probability 1-p to be degraded. From this it follows that protein production occurs in bursts with a burst size that is geometrically distributed. The experimentally observed mean burst size is five β-galactosidase proteins [7].

Using only this experimental observation, we changed the intracellular part of the above-described model to incorporate stochasticity in gene expression. This was done in the following way. We model the mRNA concentration in the cell in the same way as in the deterministic model, i.e., it only depends on the transcription and decay rate of mRNA. We now, however, interpret the mRNA concentration (if this concentration is smaller than the concentration corresponding to one molecule) as the probability that an mRNA molecule is present in the cell. We then use this probability to directly infer the frequency that a translational burst occurs, because we also know the average translation rate from the deterministic model. For example, when a certain mRNA concentration leads to a translation rate of five proteins per minute in the deterministic model and given that the average burst size equals five proteins, this now leads to a burst frequency of one burst per minute. In this way, although stochasticity is mostly determined on the mRNA level, we can still model the mRNA concentration deterministically.

Furthermore, we assume that bursts of permease and β-galactosidase are correlated, but that the number of permease molecules per burst is twice the number of β-galactosidase molecules, because the translation rate of permease is twice as large as that of β-galactosidase [21]. The unit of protein levels is now the number of molecules, and we use a minimum cell size of 8 × 10^−16^ l [22]. The units of all other variables are still mM.

Protein degradation is modeled binomially. When a cell divides, the proteins are divided randomly between the cells. Furthermore, we assume instant DNA replication, when a cell has an (arbitrarily chosen) size of 1.5 times the minimum cell size, while the cell divides at a size two times the minimum cell size. Such cell division dynamics was not taken into account in the deterministic model. In this way, we have incorporated stochasticity in gene expression with adding only one experimentally known parameter, namely the average translational burst size.

**Figure 7:**
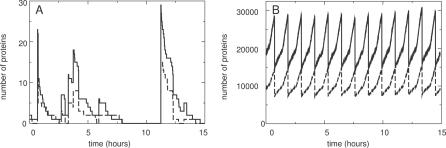
Protein Dynamics of the Stochastic Model (A) Protein dynamics for a repressed operon. The solid line indicates the number of permease molecules, the dashed line the number of β-galactosidase molecules. (B) Protein dynamics for an induced operon.

The dynamics of this model is very similar to the dynamics of previously developed models of stochastic gene expression in E. coli [11,23]. The “steady state” dynamics for a repressed and an induced operon in our model is shown in Figure 7.

#### Model for studying noise levels for lactose, IPTG, and TMG.

*Lactose.* For the simulations where we studied the amount of noise in the *lac* operon, we only considered Equations 1—6 and 9. For Equation 3 and Equation 4, we used the stochastic counterparts. We assumed a fixed growth rate of 0.01/min and (for the cAMP dynamics) zero glucose influx. We used the following parameters (if not mentioned, the parameters are equal to those listed in Table 2).

**Table 2:**
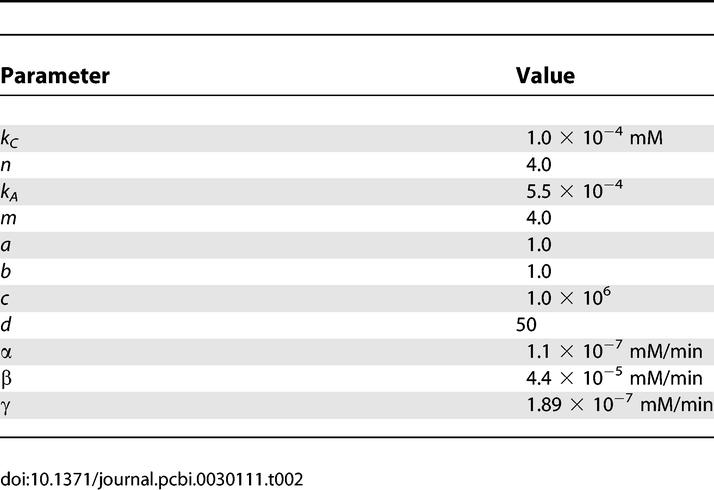
Parameters Used for Lactose

*IPTG.* IPTG is not degraded by β-galactosidase and binds directly to LacI. Therefore, the equation for promoter activity becomes

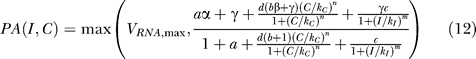



Furthermore, we add a protein-independent inducer influx term, which gives us for the IPTG dynamics

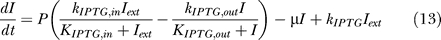



The parameters we used for IPTG are listed in Table 3 (see [13]).

**Table 3:**
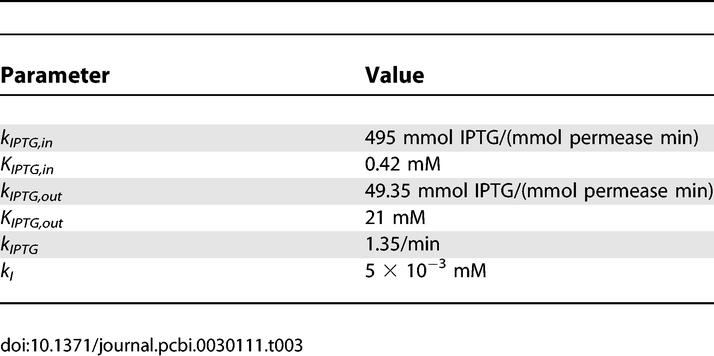
Parameters Used for IPTG

We found that, because there is no IPTG degradation and very little efflux, the external IPTG concentrations, for which the promoter function becomes induced when using the same value for *k_I_* as for induction by lactose, become unrealistically low. Therefore, we used a different value for *k_I_* than for *k_A_*. However, changing *k_I_* only shifts the promoter function to different inducer concentrations, and the noise dependence with respect to protein number remains unchanged.

*TMG.* For TMG we used exactly the same parameter values as for IPTG, except that there is no protein-independent inducer influx.

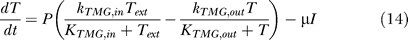



## Supporting Information

Table S1 All Parameters of the Intercellular Model and Their Values That Are Used in the Evolutionary Simulations (8 KB PDF)

## Abbreviations

CRP: cAMP receptor protein
IPTG: isopropyl β-D-thiogalactopyranoside
TMG: thiomethyl β-D-galactoside
