# Supplementary material for: The Effect of Stochasticity on the Lac Operon: An Evolutionary Perspective
Source: PLoS Comput Biol. 2007 Jun 22;3(6):e111. doi: 10.1371/journal.pcbi.0030111 (PMC1894820; doi:10.1371/journal.pcbi.0030111)
Supplement: Supplementary file 1 [file pcbi.0030111.st001.pdf]

Table S1: All model parameters with their values.

parameter	value
k_C	evolvable: initial value: 1.0×10^{-4} mM
n	evolvable: initial value: 4.0
k_A	evolvable: initial value: 5.5×10^{-4} mM
m	evolvable: initial value: 8.0
a	evolvable: initial value: 1.0
b	evolvable: initial value: 1.0
c	evolvable: initial value: 1.0×10^{6}
d	evolvable: initial value: 50
α	evolvable: initial value: 1.1×10^{-7} mM/min
β	evolvable: initial value: 2.2×10^{-5} mM/min
γ	evolvable: initial value 5.5×10^{-10} mM/min
γ_M	0.693/min
k_B	9.4 mM enzyme/(mM mRNA min)
γ_B	0.01/min
k_P	18.8 mM enzyme/(mM mRNA min)
γ_P	0.01/min
$k_{Lac,in}$	2148 mmol lactose/(mmol permease min)
$K_{Lac,in}$	0.26 mM
$k_{Lac,out}$	2148 mmol lactose/(mmol permease min)
$K_{Lac,out}$	0.26 mM
$k_{Lac-Allo}$	8460/min
$K_{m,Lac}$	1.4 mM
$k_{cat,Lac}$	9540/min
γ_L	0.15/min
$k_{cat,Allo}$	18000/min
$K_{m,Allo}$	0.28 mM
γ_A	0.15/min
$k_{cat,Glu}$	11.5 mM/min
$K_{m,Glu}$	0.45 mM
$k_{Glu,out}$	0.093/min
$k_{t,Glu}$	45 mM/min
$K_{t,Glu}$	0.015 mM
$k_{G6P,Rsp}$	34 mM/min
$K_{G6P,Rsp}$	0.5 mM
$k_{G6P,Frm}$	200 mM/min
$K_{G6P,Frm}$	20 mM
$k_{syn,cAMP}$	0.001 mM/min
$K_{syn,cAMP}$	1.0 mM/min
γ_{cAMP}	2.1/min
Y_{Rsp}	32 mM ATP/mM glucose-6-phosphate
BMC	23.5 mM/min
GC	7.28×10^{5} mM
PC	2.36×10^{6} mM ATP/mM mRNA
μ_{max}	0.0233/min
$V_{RNA,max}$	2.2×10^{-5} mM/min
